# Case of Laparotomy

**Published:** 1887-04

**Authors:** J. G. Earnest

**Affiliations:** Atlanta, Ga.


### CASE OF LAPAROTOMY.

By J. G. EARNEST, M. D, OF ATLANTA, GA.

*Read before the Atlanta Medical Society, March 1, 1887.*

The suject, ——, unmarried, æt. thirty-four years, first menstruated at fourteen. She complained of backache, constant "bearing-down pain" when on her feet, and intense ovarian neuralgia, always worse at the menstrual period. The menstrual period had been regular, except a marked increase in the amount of the flow for some time past. In spite of the fact that she had been constantly under treatment for two years by men of ability, she was still practically bed-ridden, and reduced to a skeleton, and utterly helpless.

An examination discovered a fibroid attached by a broad base, and apparently in three sections, filling up the pelvis, and firmly fixing the uterus to the floor of the pelvis. The ovaries were not to be found, on account of the position of the tumor. With a view to stopping the growth and relieving the symptoms, she was advised to have the ovaries removed. To this she readily consented, and the operation set for January 14, 1887, when, with the assistance of Doctors N. R. Harris and C. C. Greene, it was made.

She did not prove a good subject for ether (the anæsthetic used). Several times the operation was interrupted on account of the total cessation of breathing. The room had been cleaned the previous day, and scoured, and a large spray apparatus, charged with carbolic acid solution, had been playing in it for half an hour. The incision was made in the median line to within one and a half inches of the pubic symphisis, under spray. When the hand was introduced the tumor was found even larger than had been supposed. The womb was so firmly fixed on all sides that it could not be drawn up, nor could the ovaries be brought out on account of adhesions to neighboring structures. The right ovary was first gotten loose, the pedicle transfixed with a blunt needle armed with a heavy ligature of iron dyed silk that had been wet in solution of bi-chloride of mercury. The ovary was severed with scissors, and the pedicle dropped. After careful search, the left ovary was found merged in a thin-walled cyst the size of a hen's egg, the whole pushed far down behind a portion of the fibroid and firmly attached to it and neighboring structures. In spite of great care, the cyst was ruptured in getting it loose, and found to contain a blood clot and thin mucous fluid. It was necessary to extend the incision to the pubic bone, when, with great difficulty, the pedicle was reached, tied, and the ovary, with the remains of the cyst, were removed.

The toilet was made with care, and the abdominal wound closed with silver wire in the usual manner. After thorough cleansing, the wound was covered with iodoform and over this a smooth layer of borated cotton was firmly strapped. Nothing but sips of beef tea and brandy with ice was allowed for forty-eight hours; after that, milk and soup. The dressing was changed on the fourth day, when it was found dry and clean, and replaced. It was not again disturbed until the tenth day, when the stitches were removed and the wound found to be perfectly healed.

The temperature on the second day reached 102° only, for a few hours. There was no vomiting, but great nausea for the first twenty-four hours. The menstrual flow appeared on the fifth day, and continued four or five days. There has been no blood since.

The patient says she is now free from pain for the first time in two years, and is gaining flesh and much improved in appearance. The right ovary, when carefully examined, was found to contain a small cyst also.

